# Around the World in 1,475 *Salmonella* Geo-serotypes

**DOI:** 10.3201/eid2207.141678

**Published:** 2016-07

**Authors:** Céline M. Gossner, Simon Le Hello, Birgitta de Jong, Per Rolfhamre, Daniel Faensen, François-Xavier Weill, Johan Giesecke

**Affiliations:** Maastricht University Medical Centre, Maastricht, the Netherlands (C.M. Gossner);; European Centre for Disease Prevention and Control, Stockholm, Sweden (C.M. Gossner, B. de Jong, P. Rolfhamre, D. Faensen, J. Giesecke);; Institut Pasteur–World Health Organization Collaborating Centre for Reference and Research on *Salmonella*, Paris, France (S. Le Hello, F.-X. Weill)

**Keywords:** Salmonella serotypes, geographic locations, naming, serovars, geo-serotype, White-Kauffmann-Le Minor scheme, bacteria, enteric infections, salmonellae

## Abstract

It’s easy to remember *Salmonella* serotypes names, isn’t it? Surely, this is because the naming system of *Salmonella* serotypes is by far the most scientist friendly. Traditionally, most *Salmonella* serotypes have been named after geographic locations. We decided to explore the geographic locations to which *Salmonella* serotypes refer and describe some unexpected twists in the naming scheme. We found that 93% (n = 1,475) of the 1,585 serotypes could be categorized as geo-serotypes; that is, the name refers to a geographic location. The 3 countries with the most geo-serotypes are Germany, the United Kingdom, and the United States. Other serotype names refer to the name of a person, animal, tribe, or food item or are a composite of symptoms and host. The *Salmonella* serotypes naming scheme has had a valuable effect on public health microbiology, and in the current era of fast development of whole-genome sequencing, it should remain a reference.

## Abstract 2

Most *Salmonella* serotypes are named after geographic locations; a few others have surprisingly humorous origins.

What do the cities of Paris, Pisa, and Toronto have in common? Yes, all 3 are famous for their towers but what else? You don’t know? Let’s see if this will help you: what do the states of Colorado, Florida, and Ohio in the United States have in common with the 3 cities above? No idea? If we tell you *Salmonella* serotypes…. If you still don’t know, by the end of this essay you will, without a doubt, be able to answer these questions.

*Salmonella* was first isolated from a human sample in 1884 by bacteriologist Georg Gaffky and later identified as *Salmonella enterica* subspecies *enterica* serotype Typhi. The following year, the veterinary surgeon Daniel Elmer Salmon (whose name was later given to the *Salmonella* genus) and microbiologist Theobald Smith isolated *S. enterica* ser. Choleraesuis from a swine sample, while searching for the agent causing cholera in hogs (*1*). Since then, a plethora of *Salmonella* names was given to strains with new serotypes; that is, new combinations of flagellar (H) and/or somatic (O) antigens. In 1934, a first list of 44 validated *Salmonella* serotypes, called the Kauffmann-White scheme, was published (*2*).

The naming scheme of serotypes (also called serovars) evolved over time. At the beginning of the 20th century, serotype names referred to clinical syndromes either in humans (e.g., *enteritidis*, *typhi*, *paratyphi*) or in animals (e.g., *abortus-ovis, abortus-equi, typhi-murium, cholerae-suis*). The host specificity was correct for some serotypes (e.g., *abortus-ovis, abortus-equi*) but proved to be wrong for many others (e.g., *typhi-murium, cholerae-suis*) (*2*).

By the mid-1930s, Fritz Kauffmann was heading the World Health Organization Collaborating Centre for Reference and Research on *Salmonella* at the Statens Serum Institut, Copenhagen, Denmark. While there, he began to name new serotypes according to the geographic origin of the isolated strain. After Kauffmann’s retirement in 1965, Léon Le Minor became director of the World Health Organization Collaborating Centre at the Institut Pasteur, Paris, France (*3*), and he perpetuated the serotype naming scheme established by Kauffmann.

Kauffmann considered each serotype as a species and, consequently, in the old literature, the serotype names were italicized (e.g., *typhi)*. DNA-DNA hybridization, which arrived in the 1980s, showed otherwise: only 2 species (*S. enterica* and *S. bongori*) were found to be in the genus *Salmonella*. This discovery led to a long-standing debate until, in 2005, the Judicial Commission of the International Committee for Systematics of Prokaryotes made the decision to recognize the new nomenclature (*4*). Consequently, the serotype names must no longer be italicized and the first letter must be capitalized (e.g., Typhi). Names are only given to subspecies *enterica* serotypes, which represent 99.5% of all *Salmonella* strains. The remaining *Salmonella* strains are named after their antigenic formula (*2*).

Currently, >2,500 *Salmonella* serotypes have been described and listed in the “bible” of *Salmonella* serovars: the White-Kauffmann-Le Minor (WKL) scheme (*2*). Last revised in January 2007, WKL has since been completed, with 1 supplement published in 2010 (*5*) and another in 2014 (*6*). Listed in the WKL scheme are 1,585 serotypes of *S. enterica* subsp. *enterica.*

We decided to assess the geographic locations for which subspecies *enterica* serotypes are named and describe some unexpected twists in the naming scheme. First, we searched for published articles and books that recorded the first isolation of specific *Salmonella* serotypes (*7*–*12*). A large part of this exploration relied on the extensive work of the microbiologist Eckehart Kelterborn, who cataloged the history of *Salmonella* serotypes first isolations in 2 books: *Salmonella-*species: First Isolations, Names and Occurrence (*7*) and Catalogue of *Salmonella* First Isolations 1965–1984 (*8*). Then, we used the open GeoNames database (*13*) and Google Maps (*14*) to find the geographic locations corresponding with the serotype names.

Of the 1,585 serotypes of *S. enterica* subsp. *enterica* that we considered, 1,475 (93%) are geo-serotypes (i.e.,the name is associated with a geographic location); 95 (6%) have names related to a nongeographic origin (e.g., person, animal); and 15 (1%) have names of unknown origin. Geo-serotypes include serotypes for which there is a clear reference in the literature of the first isolation and link to a geographic location and for which there is no clear reference in the literature but the name is most likely associated with a geographic location with the same name. For instance, a serotype that was first described in a patient returning from France and to which was given the name of a French city was considered as a possible geo-serotype (unless contradictory information was found). The geo-serotypes were named after continents, countries, regions, islands, cities, neighborhoods, streets, gardens, rivers, lakes, and hills but also after university auditoriums, laboratories, hospitals, kibbutzim, markets, and mines.

Four geo-serotypes are linked to a broad region or continent: Africana, Antarctica, Orientalis, and Westafrica. Remarkably, serotype Antarctica was first isolated from an Emperor penguin in 1977 in the South Pole continent. The remaining 1,471 geo-serotypes can be directly associated with 1 country. The 10 countries with the most geo-serotypes are Germany (n = 181; e.g., Berlin, Brandenburg, Heidelberg); the United Kingdom (n = 167; e.g., Chester, Derby, Stanley); the United States (n = 148; e.g., Brooklyn, Chicago, Saintpaul); Nigeria (n = 74; e.g., Abuja, Ibadan, Lagos, Nigeria); France (n = 70; e.g., Avignon, Lyon, Marseille); Togo (n = 58; e.g., Adime, Lome, Djame); the Democratic Republic of the Congo (n = 58; e.g., Leopoldville, Mbandaka, Zaire); Senegal (n = 55; e.g., Dakar, Kedougou, Saboya); Sweden (n = 39; e.g., Goeteborg, Lund, Stockholm); and Ghana (n = 39; e.g., Accra, Ashanti, Victoriaborg, Goldcoast) (Figures 1, 2).

**Figure 1:**
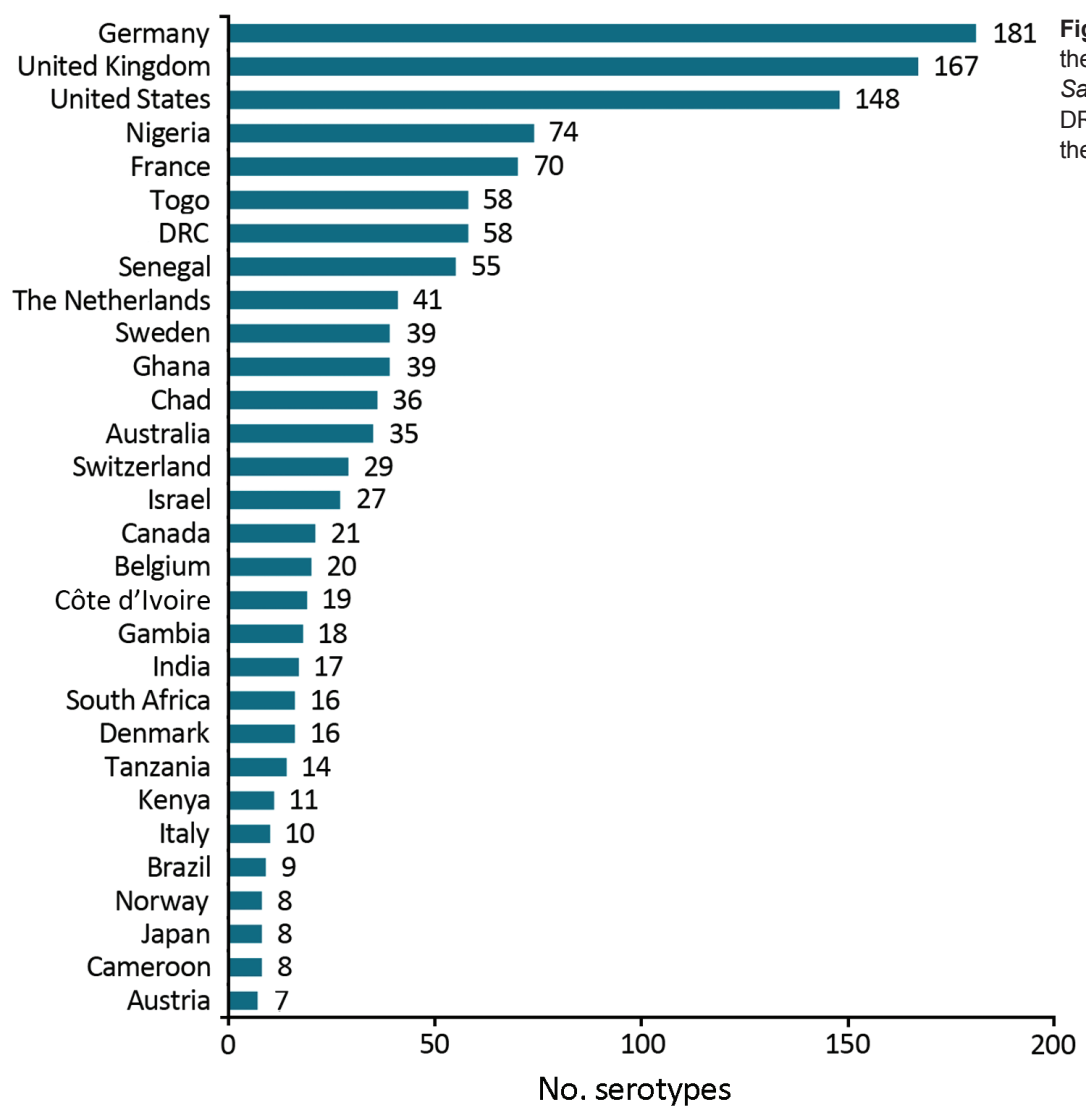
Top 30 countries with the highest number of associated *Salmonella* geo-serotypes (n = 1,259). DRC, Democratic Republic of the Congo.

**Figure 2:**
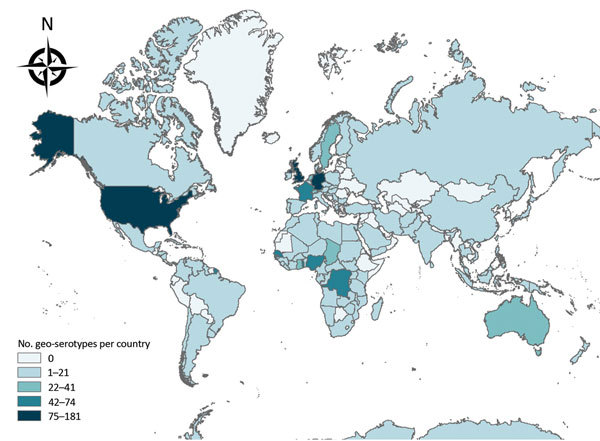
Worldwide geographic distribution of *Salmonella* geo-serotypes (n = 1,472). The geo-serotypes Africana, Orientalis, and Westafrica were excluded. Administrative boundaries copyright by Eurographics and the United Nations Food and Agricultural Organization.

Among the 1,474 *Salmonella* geo-serotypes that could be attached to a continent (Orientalis was excluded), the names of 43% are related to Europe and the names of 34% are related to Africa (Figure 2). A total of 41 geo-serotypes (3%) were named after a country, which includes current and former names of countries. Among the geo-serotypes with country names are Australia, Brazil, Bulgaria, Malaysia, and Tanzania. Singapore is represented twice, with Singapore and Sinchew, the Chinese name for Singapore. Cubana and Papuana also count as country names because they derive from Cuba and Papua New Guinea.

Fifty geo-serotypes (3%) were named after a capital city (current capital names, former capital names, and former capitals). Let’s revise our knowledge of capitals! Bangkok, Thailand; Brazzaville, Republic of Congo; Caracas, Venezuela; and Stockholm, Sweden, are current capitals. Bonn was the capital of West Germany from 1949 to 1990; Berlin is the current capital of Germany. In addition, Tananarive is the previous name of Antananarivo, the capital of Madagascar. The capital of France is named in different ways: Paris, Lutetia (the Latin name of Paris), Picpus, Vaugirard, Miromesnil, and Portedeslilas (4 metro stations), and Morillons (a street where the food safety laboratory was located). The serotype London was isolated in the city of Reading in the United Kingdom from a patient whose last name began with the letter “L.” Because the serotype Reading already existed, this serotype was named London by extension of the patient’s name.

Twenty-four states of the United States gave their names to serotypes, among which are Alabama, California, Colorado, Florida, Kentucky, Michigan, Ohio, Texas, and Utah. The states/regions of Ontario and Quebec in Canada, Nordrhein in Germany, Ashanti in Ghana, and Demerara-Mahaica in Guiana also gave their names to serotypes.

Through the years, ≈300 serotypes have been removed from the WKL scheme because they were shown to belong to other subspecies or the variant was no longer recognized. Among them, 11 referred to names of capital cities (Bern, Cairo, Buenosaires, Helsinki, Khartoum, Nairobi, Sofia, Windhoek, Zagreb, Manila, Kinshasa); 4 referred to names of countries (Angola, Argentina, Congo, Rhodesiense); and 3 referred to states of the United States (Oregon, Arkansas, Illinois). Although the serotype Buenosaires was removed from the WKL scheme, Bonariensis, the Latin name of Buenos Aires, was entered (*2*,*15*).

Instead of a location, some serotypes take their name from the name of the patient (e.g., Agbeni, Ayinde); a laboratory employee (e.g., Bamboye, Souza); an animal owner (e.g., Sarajane); the patient’s tribe (e.g., Azteca, Lokomo, Yoruba); a ship (e.g., Maron); the animal type or the food item in which the strain was isolated (e.g., Agama [lizards], Epicrates [boa], Djinten [cumin spice], Egusi [seeds]); a combination of symptoms and host (e.g., Abortusovis, Typhimurium, Typhisuis); and the Latin name of the vehicle (e.g., Aqua [water], Carno [meat], Os [bone]) . Would you think that the serotype Heron is called after the bird? That would be too easy. The strain was isolated in 1962 from a turtle by a biologist called Madam Heron (*7*).

Who says that biologists have no sense of humor? The serotype Hiduddify is named after a fictional island (*8*). The story goes as follows: In 1941, a Swede named Einar Pettersson-Skämtkvist escaped from a Japanese prisoner of war camp to arrive to the yet undiscovered island of Hiduddify, which was home of a unique ecosystem. The island was inhabited by the Rhinogradentia, mammals of a new order that were using their nose as mean of locomotion (*16*). This unique discovery was described in 1961 in a book by German zoologist Gerolf Steiner under the pseudonym Harald Stümpke. The entire story remains today a major hoax in the field of biology (*17*).

Serotype Grumpensis refers to grumpy, the name given to the owner of the guinea pig from which the strain was isolated (*7*). Ironically, the serotype Fortune refers to luck (*7*), which is certainly not the emotion felt by the person with a diagnosis of *Salmonella* infection!

In 1961, the laboratory of Colindale in the United Kingdom isolated, for the first time, serotype Egusi in egusi seeds. The same year, Colindale identified another new serotype in egusi seeds and, consequently, it was named Egusitoo (*7*). Serotype Jukestown was named by a doctor who was passionate about the juke box who lived in Georgetown, Guiana (*7*). Isolated in Chicago, the serotype Mjordan refers to the famous basketball player of the Chicago Bulls, Michael Jordan (unpub. data). Finally, other serotypes are portmanteaus or acronyms: Anfo (animal food), Ank (address not known), Ceyco (Ceylonese coconut), Chincol (Chinese egg, Colindale), Echa (egron and chamoiseau [family names of scientists who discovered this serotype]), and Inpraw (Indian prawns) (*8*).

Most of the 1,585 *Salmonella* serotypes are named after a geographic location. The list of countries that have named the most geo-serotypes correlates well with countries with strong laboratory capacities in Europe and the Americas and with countries in Africa (generally former European colonies) where some laboratory capacities (e.g., an Institut Pasteur) or close links with a laboratory in Europe had been established.

A naming scheme based on tangible names (e.g., cities, countries) has obvious advantages, such as making it easier to communicate about and pinpoint outbreaks. It is much easier to remember a label like “Agona” than the formula 1,4,[5],12:f,g,s:[1,2]. Using a naming system based on locations may, however, raise some sensitivity. National or local authorities may not appreciate the association of their area with a pathogen, especially when large foodborne outbreaks are highly publicized by the media. The same applies for serotype names based on the name of a food product. For instance, outbreaks of *S*. *enterica* ser. Djinten (cumin spice) are certainly not a good selling pitch for cumin producers/distributors. Therefore, serotype names should be interpreted with caution, and consumers should be reminded that no direct relationship exists between the serotype name and the prevalence of cases in the specific location or by the consumption of a specific product. The likelihood of acquiring *S. enterica* ser. Heidelberg infection in the city of Heidelberg, Germany, is probably no higher than the chance of acquiring the same infection in Miami, Florida, USA. Studying the correlation between serotypes’ names and places of infection could be intriguing.

The affiliation of a new variant to a previously recognized serotype may have more implications than a simple name attribution. Although the monophasic variant 1,4,[*5*],12:i:- emerged in the 2000s, only in 2010 was it officially recognized as part of serotype Typhimurium by the European Union (*18*). Because of its atypical antigenic formula, this variant avoided for years all European Union laws applying to *S*. *enterica* ser. Typhimurium. It is certainly a proof of natural selection against European Union legislation.

The introduction of DNA-based methods targeting neutral markers such as multilocus sequence typing demonstrated that most of *Salmonella* serotypes span multiple, genetically unrelated clusters (*19*). Therefore, as multilocus sequence typing and, ultimately, sequence-based typing methods based on entire genomes are more discriminatory than serotyping, the serotype-based nomenclature will ideally be complemented by a genome sequence-based typing scheme (*19*). A genome type/serotype dictionary should be developed to maintain the link with the serotyping nomenclature, to continue building on >80 years of accumulated data, and to ensure a smooth transition for countries or regions in the world that will not switch to whole-genome sequencing as fast as others.

To answer the question posed at the beginning of this article—indeed, Paris, Pisa, Toronto, Colorado, Florida, and Ohio have all given their name to *Salmonella* serotypes. As promised, the material provided in this short review on the *Salmonella* naming scheme will help you interpret and decipher *Salmonella* names.
